# Intranasal Trans-Sialidase Vaccine Mitigates Acute and Chronic Pathology in a Preclinical Oral Chagas Disease Model

**DOI:** 10.3390/vaccines12101171

**Published:** 2024-10-15

**Authors:** Maria Florencia Pacini, Camila Bulfoni Balbi, Brenda Dinatale, Cecilia Farré, Paula Cacik, Florencia Belén Gonzalez, Iván Marcipar, Ana Rosa Pérez

**Affiliations:** 1 Instituto de Inmunología Clínica y Experimental de Rosario (IDICER-CONICET), Rosario 2000, Argentina; pacini@idicer-conicet.gob.ar (M.F.P.); bulfonibalbi@idicer-conicet.gob.ar (C.B.B.); dinatale@idicer-conicet.gob.ar (B.D.); farre@idicer-conicet.gob.ar (C.F.); gonzalez@idicer-conicet.gob.ar (F.B.G.); 2 Centro de Investigación y Producción de Reactivos Biológicos (CIPReB), Facultad de Ciencias Médicas, Universidad Nacional de Rosario, Rosario 2000, Argentina; 3 Laboratorio de Tecnología Inmunológica, Facultad de Bioquímica y Ciencias Biológicas, Universidad Nacional del Litoral, Santa Fe 3000, Argentina; paula.cacik@gmail.com (P.C.); imarcipr@fbcb.unl.edu.ar (I.M.)

**Keywords:** oral Chagas disease, chronic Chagas cardiomyopathy, *Trypanosoma cruzi*, trans-sialidase, nasal vaccine, c-di-AMP

## Abstract

Chagas disease, caused by *Trypanosoma cruzi*, leads to severe complications in 30% of infected individuals, including acute myocarditis and chronic fibrosing cardiomyopathy. Despite the significant burden of this disease, there is currently no licensed vaccine available to prevent it. This study aimed to evaluate the mucosal and systemic immunogenicity as well as the prophylactic efficacy of a mucosal vaccine candidate and its impact on both acute and chronic cardiomyopathy. The results showed that the nasal administration of trans-sialidase (TS) plus c-di-AMP (TS+A) vaccine elicited a NALT expression of IFN-γ, IL-17a and IL-4 mRNA as well as a nasal-specific production of IgA. An in vivo challenge with TS also triggered increased proliferation of lymphocytes from the NALT, sentinel cervical lymph node, and spleen. TS+A immunization increased the plasma levels of Th1/Th2/Th17 cytokines and elicited an evident cellular response by which to judge enhanced delayed-type hypersensitivity responses following a TS footpad challenge. After oral infection, TS+A-vaccinated mice showed significantly reduced parasitemia and parasite load in the heart, muscles and intestines, while markers of hepatic and muscle damage as well as clinical manifestations of acute infection were strongly diminished. TS+A also attenuated acute myocarditis and the expression of inflammatory markers in the heart. The protection conferred by TS+A extended into the chronic phase, where it resulted in a clear reduction in chronic myocarditis, fibrosis and functional electrocardiographic abnormalities, associated with a decreased expression of the pro-fibrotic TGF-β. These results revealed that it is possible to develop a mucosal vaccine against *T. cruzi* based on TS and c-di-AMP that is capable of reducing the development of Chagas cardiomyopathy, the hallmark of Chagas disease.

## 1. Introduction

Chagas disease, caused by the protozoan parasite *Trypanosoma cruzi*, continues to be a major public health challenge in Latin America. According to the World Health Organization, approximately 7 million people are currently infected across 21 countries in the region, with around 70 million individuals at risk of contracting the disease [1]. The infection leads to severe complications, including acute myocarditis and chronic fibrosing cardiomyopathy, which can result in heart failure, arrhythmias and other life-threatening cardiac issues in about 30% of chronic cases [2,3,4,5].

Although vector-borne transmission is the most recognized route, *T. cruzi* can also spread through congenital transmission, organ transplantation, blood transfusion and, increasingly, through oral transmission [4,6,7]. Evidence suggests that oral infection often presents with more severe symptoms compared to vector-borne transmission, even in adults. The past few decades have seen a marked increase in cases of acute Chagas disease linked to oral infection, contributing to a rise in fatalities from acute cardiomyopathy [8,9].

Experimental vaccines against Chagas disease, typically administered via subcutaneous, intramuscular, or intradermal routes, aim to elicit robust systemic humoral and cellular responses to combat the parasite effectively. However, these studies showed no evidence of enhancing mucosal immunity, which, alongside the skin, is a primary entry point for *T. cruzi*. The potential of mucosal immunization has been relatively overlooked in Chagas disease research, despite its importance. Notably, the conjunctival mucosa, associated with the Romaña sign, and the buccal mucosa, often leading to facial edema in oral cases, are well-documented sites of natural human infection [10,11]. Experimental evidence further underscores the significance of mucosal entry points: the naso-maxillary region is a critical site for parasite entry following oral infection in mice, where the parasite persists and predominates for extended periods [12]. Similarly, studies on conjunctival mucosa infection revealed that parasites can drain through the nasolacrimal duct and subsequently infect the nasal cavity epithelium [13]. These findings, alongside previous research from our group [14], suggest that intranasal immunization with *T. cruzi*-derived antigens could effectively prevent parasite dissemination, regardless of the entry site (be this oral, other mucosal surfaces, or even the skin). This approach could offer protection against both acute and chronic disease by inducing a comprehensive systemic and mucosal immune response.

Some members of the trans-sialidase (TS) superfamily of *T. cruzi* proteins have been widely utilized as immunogens in various vaccine strategies, yielding promising results [15,16]. The active TSs, referred to as TS-Group I, are particularly immunodominant antigens in both mice and humans. These proteins exhibit enhanced efficacy as vaccine immunogens when their enzymatic activity is inhibited and the Shed Acute Phase Antigen (SAPA) domain is deleted, as the SAPA domain often acts as a decoy for the immune system [15]. Consequently, developing a recombinant vaccine based on an active TS fragment, in which the immunodominant SAPA repeats are removed while preserving the protein’s overall structure, represents a promising strategy for enhancing vaccine efficacy against *T. cruzi*.

In this context, we previously demonstrated that administering various recombinant fragments from active TS-based formulations via the oral or nasal routes offers distinct advantages over other methods, as it can elicit specific immune responses at both mucosal and systemic levels [14,17,18]. Importantly, while several preclinical vaccines tested so far have significantly reduced parasitemia during the acute phase, none have achieved sterilizing immunity, partly due to the complex life cycle of the parasite. Given the challenge of attaining sterilizing immunity, developing a vaccine that effectively reduces *T. cruzi* loads, prevents disease progression or mitigates the severity of cardiac complications is crucial. Such a vaccine would improve the quality of life for individuals affected by Chagas disease by decreasing morbidity and mortality associated with cardiac issues, ultimately leading to a substantial reduction in the incidence of chronic chagasic cardiomyopathy and a significant alleviation of the disease burden in endemic regions.

Chronic infection with the parasite *T. cruzi* leads to an inflammatory cardiomyopathy, resulting in scarring and significant alterations in cardiac structure, clinically referred to as Chronic Chagasic Cardiomyopathy (CCC) [19,20]. The invasion of parasites into cardiomyocytes recruits inflammatory cells, including macrophages, neutrophils and CD4+ and CD8+ T cells, resulting in an inflammatory environment that is involved in the development of cellular hypertrophy [21,22]. Cytokines play a crucial role in regulating the immune response during the chronic phase of Chagas disease, aiding in infection control while also contributing to myocardial dysfunction [23]. While high levels of mRNA for both pro- and anti-inflammatory cytokines have been observed in mononuclear cells from patients chronically infected with *T. cruzi* [24], research indicates that cytokine production varies among patients with different clinical manifestations of the disease [24]. Inflammatory cytokines, like IFN-γ and TNF-α, are associated with more severe clinical outcomes and the progression of CCC, while cytokines with a modulatory profile are linked to the maintenance of the indeterminate form, where cardiac and digestive functions remain largely unaffected. These findings underscore the importance of balancing pro- and anti-inflammatory immune profiles in the context of Chagas disease, suggesting that eliciting a balanced Th1/Th2/Th17 response could help control persistent parasites without exacerbating inflammatory or fibrotic damage in the host.

This study aimed to evaluate the immunogenicity and prophylactic efficacy of a nasally administered vaccine against oral *T. cruzi* infection, as well as its impact on both acute and chronic cardiomyopathy. The vaccine formulation tested included N-terminal recombinant fragments of a Group I trans-sialidase (TS), which lacks enzymatic activity and the immunodominant SAPA antigen, combined with bacterial dinucleotide c-di-AMP, known for its immunostimulatory properties in the nasal mucosa [25,26,27,28].

## 2. Materials and Methods

### 2.1. Expression and Purification of TS Recombinant Antigen

The N-terminal TS fragment was selected, cloned and expressed in *E. coli* as previously described by our group [29]. This fragment lacks enzymatic activity and the SAPA antigen and contains T- and B-epitopes predicted by bioinformatics [15]. Later, fragment purification was performed by affinity chromatography on Ni2+-NTA-agarose columns (Invitrogen TM, Waltham, MA, USA) with an imidazole/imidazole gradient of 8 M urea. All fractions were collected and dialyzed against PBS/0.5 M urea and quantified by the bicinchoninic acid method. The presence of lipopolysaccharide (LPS) in the recombinant protein was determined using the Limulus Amebocyte Lysate (LAL test) kit (Genscript, Piscataway, NJ, USA). The LPS levels were <10 endotoxin units (EU)/mg protein. The purity of the protein was checked using SDS-PAGE. The N-terminal fragment was indexed in GenBank with the accession number MZ215730.

### 2.2. Mice

BALB/c female mice (6–8 weeks old) were obtained from the animal facilities at the Facultad de Ciencias Veterinarias de la Universidad Nacional de La Plata (LAE-FCV-UNLP) and kept in HEPA-ventilated cages at the facilities of the Centro de Investigación y Producción de Reactivos Biológicos, Facultad de Ciencias Médicas, Universidad Nacional de Rosario (CIPReB-FCM-UNR), both in Argentina. All protocols for animal studies were approved by the Institutional Animal Care and Use Committee (Res. Nro: 6698/2014 and No: 2958/2018), according to the institutional guidelines and carried out following the National Institutes of Health’s ‘Guide for the Care and Use of Laboratory Animals’.

### 2.3. Immunization and Infection Protocol

Briefly, mice (4–7/group) were immunized intranasally with 3 doses (1 dose every 2 weeks) of each formulation containing: (a) a vehicle (a saline solution, SS group); (b) 10 µg of N-terminal TS fragment diluted in the saline solution without any adjuvant (TS group); (c) 10 µg of N-terminal TS fragment plus 5 µg of c-di-AMP (TS+A group); and (d) an adjuvant alone as control group (A group). Two weeks after the last immunization, immunized animals were infected orally with a non-lethal dose of 2500 bloodstream trypomastigotes of Tulahuen strain (DTU-VI), maintained by serial passages in Cbi suckling mice (CIPREB-FCM-UNR). Parasitemia was monitored every 7 days by the examination of 5 mL blood samples via direct microscopic evaluation, as previously described by our group [14].

### 2.4. Determination of Specific Antibodies in Plasma and Nasal Lavages

Plasma was obtained by centrifugation at 5000 rpm of heparinized blood, which was subsequently stored at −20 °C until total IgG, IgG2a and IgG1 evaluation. To evaluate IgA in nasal secretions, nasal lavages were performed following the protocol reported by Cho and col [30]. Briefly, it was obtained by retrograde infusion through the trachea with 0.3 mL of a cocktail containing protease inhibitors (cOmplete™ Protease Inhibitor Cocktail, Roche, Indianapolis, IN, USA) and stored at −80 °C until use. For the detection of TS-specific antibodies, ELISA microplates (Nunc-Immuno MaxisorpTM, Thermo, Waltham, MA, USA) were coated with the selected N-terminal fragment (0.5 µg/well) diluted in a carbonate–bicarbonate buffer (0.05 M; pH 9.6). The procedure for determining plasma and nasal lavage antibodies was performed following the same protocol reported previously by our group [14]. Briefly, samples were read at 450 nm with a correction to 545 nm in an ELISA reader (Epoch Biotek Instruments, Santa Clara, CA, USA) after incubation with 100 µL of ready-to-use trimethylbenzidine (Wiener Lab, Rosario, Argentina) followed by the addition of 50 µL of 2N H_2_SO_4_. (Wiener Lab, Argentina). Optical density (OD) values for Ig measurement were adjusted by subtracting the OD reading from the diluent (blank).

### 2.5. Delayed Type Hypersensitivity Test

A delayed-type hypersensitivity test (DHT) was performed by intradermal challenge with TS (5 μg of TS fragment) in the hind footpad of mice 15 days after the last immunization. Footpad swelling was measured with a digital caliper at 0, 24, 48 and 72 h post-challenge, as informed previously [14].

### 2.6. Lymphocyte Culture and Flow Cytometry

After completing the immunization schedule, NALT, cervical lymph node (CLN) cells and splenocytes were obtained by mechanical organ disaggregation in PBS+3%FBS (Gibco, Waltham, MA, USA). Subsequently, 1 × 10^6^ cells/wells were cultured in 48-well plates (GBO) in DMEM medium supplemented with 10% FBS (Gibco), 2% penicillin (100 g/mL, Sigma, Burlington, MA, USA) and streptomycin (100 U/mL, Sigma), either alone or stimulated with TS (10 μg/well). After 16 h of culturing, cells were incubated with anti-FcγII/III-R antibodies and stained with anti-CD4/PerCP, anti-CD8/PE, anti-B220/APC–Cy7 and anti-Ki67/FITC (all from BD Pharmingen, San Diego, CA, USA) to determine lymphocyte proliferation. Samples were acquired using a BD FACSAria-II cytometer, with a minimum of 1 × 10^5^ events acquired for each sample. FlowJo v10 Software (Beckton–Dickinson, Franklin Lakes, NJ, USA) was used for sample analysis.

### 2.7. Quantification of Plasma Cytokines

Plasma interleukin (IL)-2, IL-4, IL-6, IFN-γ, TNF-α, IL-17A and IL-10 levels were quantified in samples obtained at −1, 17- and 111-days post-infection (pi) by flow cytometry using the BD Cytometric Bead Array (BD Biosciences, San Jose, CA, USA), following the manufacturer’s instructions. Samples were acquired on a BD FACSAriaII flow cytometer (Beckton–Dickinson, USA) and the concentrations of the cytokines of interest were determined using the BD FACSDiva v9.0 software.

### 2.8. NALT and Heart Cytokine Profiling by RT-qPCR

The cytokine profile was analyzed in the NALT (day −1 pi) and heart (days 17 and 111 pi). Briefly, the NALT was isolated by carefully cutting the mice’s upper palate with a scalpel, following the inner contour of the incisors and molars, and then gently peeling it off with forceps [31]. The cells were extracted with TRI-Reagent^®^. Additionally, half a heart was removed and mechanically disrupted in TRI-Reagent^®^. The RNA was then reverse transcribed into cDNA using RevertAid Reverse Transcriptase (Thermo–Fisher Scientific, USA). Real-time PCR was performed on StepOnePlus equipment (Applied–Biosystems, Waltham, MA, USA) using Mix-5x-HOT-FIREPol^®^EvaGreen^®^ qPCR-Mix Plus with ROX (Solis–BioDyne, Tartu, Estonia). The amplification program included an initial activation step at 95 °C for 15 min, followed by denaturation at 95 °C for 15 s, an annealing temperature between 59°/62 °C, and finally an elongation at 72 °C for 20 s, for 40 cycles. Fluorescence was measured after each extension step and the specificity of the amplification was assessed via melting curve analysis. The GAPDH gene was used as a control to normalize the mRNA samples. Relative gene expression levels were calculated using an ad hoc standard curve for each gene. Amplification efficiencies were identical or similar between genes of interest and controls. The primers used were in Supplementary Table S1.

### 2.9. Clinical Score

To estimate the clinical impact of vaccine protective efficacy, we used a clinical scoring system developed by our group for non-lethal models of *T. cruzi* infection [14]. Briefly, the individual score was computed as the sum of the pre-established values for each clinical sign, as follows: absence of signs (0), piloerection (1), hunched posture (1.5), ocular involvement (2), decreased locomotor activity (2.5) and diarrhea (3). The overall score for each group/day was calculated as the average of individual scores. Finally, the cumulative score of each group was determined as the area under the curve (AUC).

### 2.10. Determination of Muscle and Liver Damage in the Acute and Chronic Phases

After infection, the activity of enzymes indicative of muscle and liver damage was assessed in fresh plasma: creatine kinase (CK), glutamate oxaloacetate transaminase (GOT) and glutamate pyruvate transaminase (GPT). These were measured using spectrophotometric tests, following the manufacturer’s instructions (Roche).

### 2.11. Detection of Parasite Burden in Target Tissues

Heart, skeletal muscles and small intestines were collected for DNA extraction, following the method described previously [32]. Briefly, total DNA was extracted from each sample after tissue disaggregation with a lysis buffer (10 mM Tris-HCl pH = 7.6; 0.1 M NaCl; 10 Mm EDTA; 0.5% SDS, 300 µg/mL proteinase K from Sigma). The samples were then heated for 2 h at 55 °C, and extracted twice with a phenol–chloroform–isoamyl alcohol mixture (25:24:1) (Sigma Chemical Co., St. Louis, MO, USA). Cold ethanol (AAPER Alcohol and Chemical Co., Shelbyville, KY, USA), twice the volume of the extracted sample, was then added and the samples were placed at −80 °C for o.n.. Samples were centrifuged for 30 min at 13,000 rpm and washed with 70% ethanol, vacuum dried and then resuspended in sterile water. DNA samples were adjusted to a final concentration of 25 or 125 ng/μL and were used as a template for the qPCR reactions using the specific primers for *T. cruzi*: kDNA was amplified using S36 (5′GGT TCG ATT GGG GTT GGT G3′) and S67rev (5′GAA CCC CCC TCC CAA AAC C3′) primers. PCR reactions were performed using a HOT FIREPol EvaGreen qPCR Mix Plus kit (Solis Biodyne, Tartu, Estonia) with a StepOneTM Real-Time PCR Systems (Applied Biosystems) instrument. The parasite load was expressed as equivalent parasites in 50 ng of murine DNA.

### 2.12. Histopathology

Footpads (−1 day pi), hearts and sections of the quadriceps muscle (both at 17 and 111 days pi) were removed, fixed in 4% formalin and then embedded in paraffin. Then, 5 μm sections were stained with hematoxylin/eosin for an evaluation of inflammatory infiltrates and cardiac and skeletal tissue damage, similar to that described previously by our group [14]. Additional sections of heart and skeletal muscles were stained with picrosirius red to evaluate tissue fibrosis. Fibrosis is classified based on the size and number of cardiac fibers affected and then determined as mild, moderate or severe. Taking these findings into account, a score was designed to quantify it. The slides were analyzed blindly by an expert pathologist, who was not privy to the data.

### 2.13. Electrocardiograms

At 111 days pi, mice were anaesthetized with a 50%:10% Ketamine–Xylazine solution (acquired from Holliday–Scott S.A., Béccar, Argentina and PharmaVet, Carole Park, Australia, respectively) and their cardiac function was subsequently monitored by electrocardiography. The recording of the electrocardiograms (ECG) was performed using an electrocardiograph specially designed for laboratory animals (Cardiocom, San Luis, Argentina) and equipped with the CC7DerS-Vet 1.0.4 software, supplied by the equipment manufacturer, which allows for the evaluation of the heart rate (Fc) of each mouse and different parameters of cardiac functionality, such as the P wave, the PR interval, the PR segment, the QRS complex and the QT interval. As the value of the QT interval varies with the recorded Fc (decreases at high Fc and increases at low Fc), its value was corrected by applying the Mitchell’s formula [33] to obtain the corrected QT interval (QTc). Leads I and II were analyzed, and all alterations that showed some type of arrhythmia was recorded.

### 2.14. Statistical Analysis

Data were analyzed using non-parametric tests; namely the ANOVA Kruskal–Wallis test (for k groups > 2), followed by the Mann Whitney U test (for comparisons between two groups). Data were shown as mean ± SEM or median/range depending on variable distribution. All analyses were performed using the GraphPad Instat 4.0 software (GraphPad, San Diego, CA, USA). Differences between groups were considered significant when the *p*-value was <0.05.

## 3. Results

### 3.1. TS+A Recombinant Vaccine Elicits Both Mucosal and Systemic Immunogenicity

**Figure 1:**
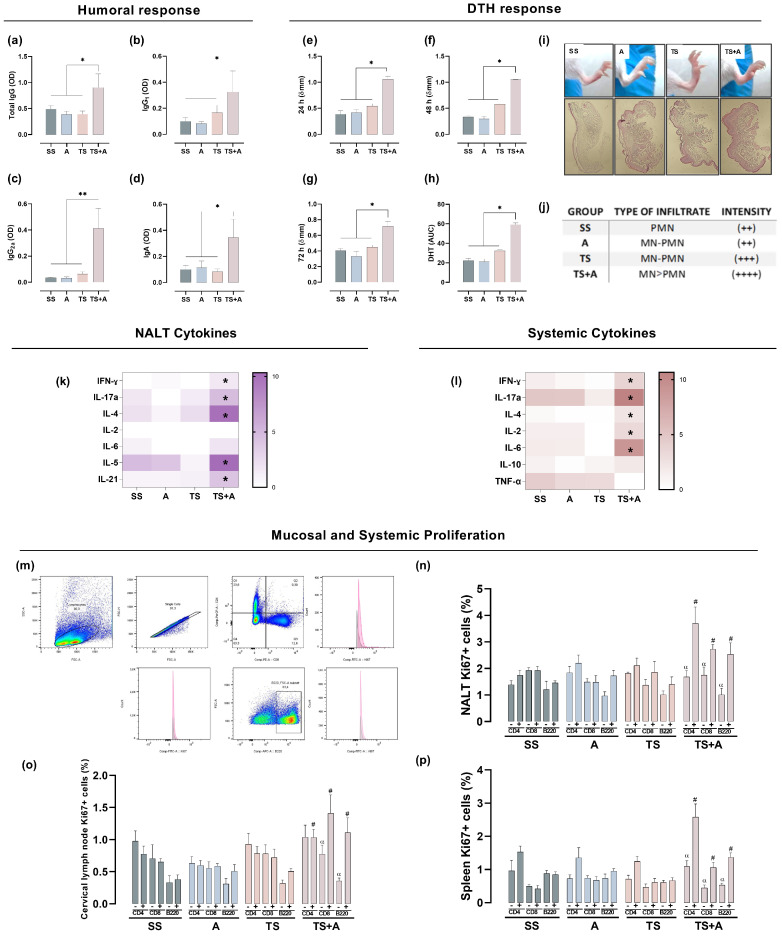
**Mucosal and systemic immune response triggered by intranasal immunization 15 days after vaccination.** TS-specific systemic (**a**–**c**) and mucosal (**d**) humoral response. Delayed type hypersensitivity -DTH- test along 72 h (**e**–**g**), where level of swelling was expressed as delta (Δ). (**h**) The area under the curve (AUC) from 0 to 72 h after DTH. (**i**) Representative images of DTH after 24 h. (**j**) Inflammation intensity and type of infiltration after 24 h of challenge (PMN: polymorphonuclear and MN: mononuclear). (**k**) Heatmap of NALT mRNA expression of cytokines. (**l**) Heatmap of plasma cytokine concentrations. The levels of significance are defined as follows: * *p* < 0.05 and ** *p* < 0.01. (**m**) Gating strategy to estimate ex vivo lymphoid cell proliferation after restimulation with TS for 16h in: (**n**) NALT cells, (**o**) cervical lymph node cells and (**p**) splenocytes. The levels of significance are defined as follows: α = *p* < 0.05 vs. corresponding stimulated cells, # = *p* < 0.05 compared to stimulated cells of the rest groups.

Fifteen days after completing the immunization schedule, the specific humoral immune response towards the N-terminal fragment of the catalytic domain of TS was analyzed, whether accompanied or not by the c-di-AMP (A) adjuvant (Figure 1). As shown in Figure 1a, animals immunized with the formulation containing TS+A showed a significant increase in total IgG compared to the groups receiving the remaining formulations. The TS+A group also showed a substantial systemic response in terms of IgG2a and IgG1, with an IgG2a/IgG1 ratio ≈ 1, (Figure 1b,c). In addition, the evaluation of nasal secretions indicated that mice immunized with TS+A presented increased levels of TS-specific IgA at the immunization site compared to the other groups. Animals vaccinated with the TS fragment alone showed similar values to those of SS and A (Figure 1a–d). To assess in vivo cell-mediated immunity, a delayed-type hypersensitivity (DTH) test was conducted on the footpads of mice. A pronounced swelling was revealed at 24 and 48 h post-challenge in the TS+A group (Figure 1e,f). Although it decreased by approximately 40% after 72 h, it remained consistently elevated in the TS+A group compared to the others (Figure 1g). The DTH response induced in animals immunized with the TS fragment alone did not differ significantly from that observed in the SS group, indicating that the presence of di-AMP-c is necessary to induce the immunogenicity of the fragment. As seen in Figure 1h, the overall cellular response, measured as the area under the curve (AUC) from 0 to 72 h, confirmed that only mice sensitized with TS+A triggered an evident specific cellular response against the N-terminal recombinant fragment. Histological analysis of the footpads at the peak of reactivity revealed an increase in immune cell infiltration, with a greater intensity observed in the TS+A group (Figure 1i,j). The infiltration mainly comprised polymorphonuclear cells in the SS group, while in contrast, mice immunized with TS, A and TS+A exhibited a mixture of mononuclear and polymorphonuclear cells (Figure 1j). Notably, the TS+A group displayed a predominantly mononuclear infiltration compared to the other groups. Mice administered with only the vehicle (without antigenic stimulus) showed no infiltration of any kind (Figure 1i,j). Globally, results of enhanced DTH reactivity recorded in the TS+A group showed a consistent clinical–histological concordance.

In the mucosa, the development of specific immune responses depends not only on the nature of antigenic stimulation but also on specialized inductive structures, such as NALT, and the subsequent expression of proinflammatory and immunoregulatory cytokines. Hence, to determine whether the formulation containing TS+A triggers in the NALT a particular cytokine profile, a wide range of cytokines associated with diverse immune patterns of response was evaluated using RT-qPCR. As can be observed in Figure 1k, mice immunized with TS+A displayed locally a significant increase in the expression of IFN-γ, IL-4, IL-5, IL-21 and IL-17a mRNA, compared to the SS, TS and A groups, while no differences were found in IL-2 and IL-6 between the groups. Similarly, plasma samples were evaluated to detect whether nasal immunization elicits a specific cytokine pattern. As shown in Figure 1l, mice immunized with TS+A presented a slight but detectable increase in circulating IFN-γ, IL-4, IL-17A, IL-6 and IL-2 compared with the rest of the groups. Although a tendency to increase IL-10 was also observed in the TS+A mice, this was not significant when compared with the rest of the groups (Figure 1l). Conjointly, data showed that TS+A formulation triggers a mucosal and systemic pattern of cytokines linked to an improved anti-*T. cruzi* immunogenic response.

Lastly, the proliferative potential of responding TS-specific lymphocytes was evaluated upon ex vivo restimulation with TS in NALT, CLN and spleen cells, following the gating strategy shown in Figure 1m. The proportion of CD4+, CD8+ and B220+ lymphocytes proliferating in response to TS (detected as Ki67+ cells) was similar to that of the non-stimulated cells in the SS, TS and A groups (Figure 1n–p). The proliferative response to TS was notably more evident in NALT, CLN and spleen lymphocytes from mice vaccinated with TS+A when compared to the other groups. Similarly, in the cells from the TS+A group exposed to TS, the proliferative response was higher than the unstimulated cells, except for the CLN CD4+ T cells (Figure 1n–p). These results showed that the TS+A formulation may trigger in both inductive and effector mucosal sites, as well as in a systemic secondary lymphoid organ, a critical recall response for controlling *T. cruzi*.

### 3.2. Prophylactic TS+A Immunization Confers Protection during Acute Oral T. cruzi Infection

**Figure 2:**
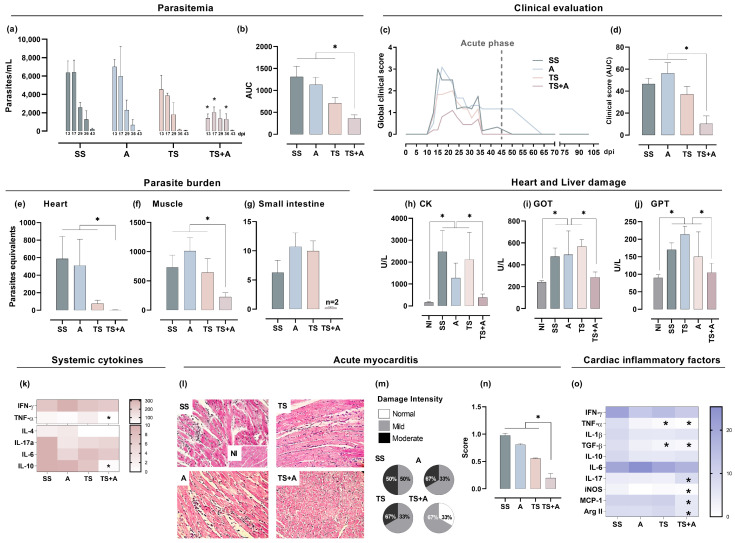
**Vaccine efficacy in the acute phase.** (**a**) Parasitemia. (**b**) Area under the curve (AUC) of the parasitemia from day 0 to day 43 pi. (**c**) Clinical evolution recorded daily, expressed as a clinical score (data are expressed as median values of each group/day). (**d**) Global clinical involvement of each group, expressed as AUC. (**e**–**g**) Tissue parasite burden at 17 dpi (n = 5/group, except for TS+A, with a n = 2). (**h**–**j**) Plasma activity of heart, muscle and liver damage-associated enzymes. (**k**) Heatmaps of plasma cytokines after 17 dpi. Acute myocarditis at day 17 pi: (**l**) Representative images of cardiac tissues (40× magnification); (**m**) Infiltration intensities: pie charts illustrate the proportion of animals within each group that exhibited each type of inflammatory infiltration. (**n**) Inflammatory score estimates the degree of tissue damage in each group based on the number and intensity of inflammatory infiltrations. (**o**) Heatmap showing heart cytokine expression during the acute phase. * *p* < 0.05.

To assess the efficacy of the prophylactic nasal administration of TS-based formulations, mice were orally challenged with a sub-lethal dose of *T. cruzi* 15 days after completing the immunization schedule. As shown previously [14], a sub-lethal model was selected to simulate what occurs in humans, where the infection is lethal in only a small proportion of infected individuals. First, the number of blood trypomastigotes and the clinical repercussion displayed during the acute phase were examined. Parasitemia peaks were recorded between 2 and 3 weeks pi and decreased progressively until they disappeared completely after 6 weeks pi, these being significantly lower in the TS+A group than in the rest of the groups (Figure 2a). The improved control of circulating forms of *T. cruzi* accomplished in TS+A vaccinated mice was reflected by the three-fold reduction in the cumulative parasitemia curve (AUC) compared to the SS group (Figure 2b). In infected animals, clinical signs began to appear around day 10 pi and disappeared after 60 days. However, animals immunized with TS+A exhibited a significant reduction in morbidity compared to those receiving other formulations, given that clinical signs reverted after 36 days pi (Figure 2c). A cumulative assessment of the clinical impact of nasal vaccination showed a marked attenuation of acute signs of infection in TS+A immunized animals compared to the rest (Figure 2d).

Given the nature of the parasite strain used in this study (the Tulahuen strain is reticulotropic but also myotropic) and the route of infection selected, a search for intracellular parasites was achieved in the heart, skeletal muscles and small intestines of the immunized animals. In a similar way to what was observed with blood trypomastigotes, parasite burden in target tissues was strongly diminished in the TS+A immunized mice (Figure 2e–g). In addition, since parasitism can lead to tissue damage, the activity of enzymes acting as markers of heart, muscle and liver tissue damage were also analyzed in immunized mice. An evident activity of creatinine kinase (CK), glutamate oxaloacetate transaminase (GOT) and glutamate pyruvate transaminase (GPT) (Figure 2h–j) was observed during the peak of parasitemia in the SS, TS and A groups. Conversely, in the TS+A group, although CK only showed a slight increase, globally they did not differ from those observed in the NI control group. These results indicate the administration of TS+A prevented cardiac, muscle and liver damage during the peak of parasitemia. In addition, when evaluated for other systemic markers of inflammation, it was observed that TNF-α and their counterregulatory cytokine IL-10 were significantly decreased in TS+A immunized mice concerning other groups, while no differences were found in terms of IFN-γ, IL-4, IL-6, IL-2 and IL-17A (Figure 2k). Moreover, histopathological studies of the heart indicated that the TS+A vaccine provides cardiac protection, given mice immunized with TS+A showed little or no proportion and size of inflammatory foci in comparison to the other groups (Figure 2l,m). Moreover, 33% of TS+A immunized and infected animals showed no evidence of myocarditis at day 17 pi (Figure 2m). These findings were reflected in the lowest score of histological findings recorded in the TS+A group (Figure 2n). Histological findings in the skeletal muscle samples were similar to those in the heart samples, showing large infiltrations affecting a large number of myocytes in the SS group, and, on the contrary, small foci and attenuated damage in the TS+A group (Supplementary Figure S1). There is convincing evidence that cytokine networks can influence the clinical outcome of CD, for that we evaluated the levels and expression of various cytokines. Furthermore, hearts from TS+A vaccinated mice showed lower expression levels of TNF-α, TGF-β, MCP-1, iNOS and Arg-II, while displaying contrasting levels of IL-17, compared to the SS group at day 17 pi (Figure 2o). A similar behavior was also observed in the TS group regarding TNF-α and TGF-β (Figure 2o). No significant differences in IL-10, IFN-γ, IL-1β, IL-6 and iNOS expression were found among the groups (Figure 2o). These findings collectively demonstrate that nasal mucosa administration of TS+A, exhibits not only high efficacy in parasite control but also in mitigating both inflammatory markers and tissue damage, thereby contributing to a favorable course during the acute phase following oral *T. cruzi* infection.

### 3.3. Prophylactic Immunization with TS+A Formulation Attenuates Chronic Chagasic Myocarditis and Heart Function Abnormalities

**Figure 3:**
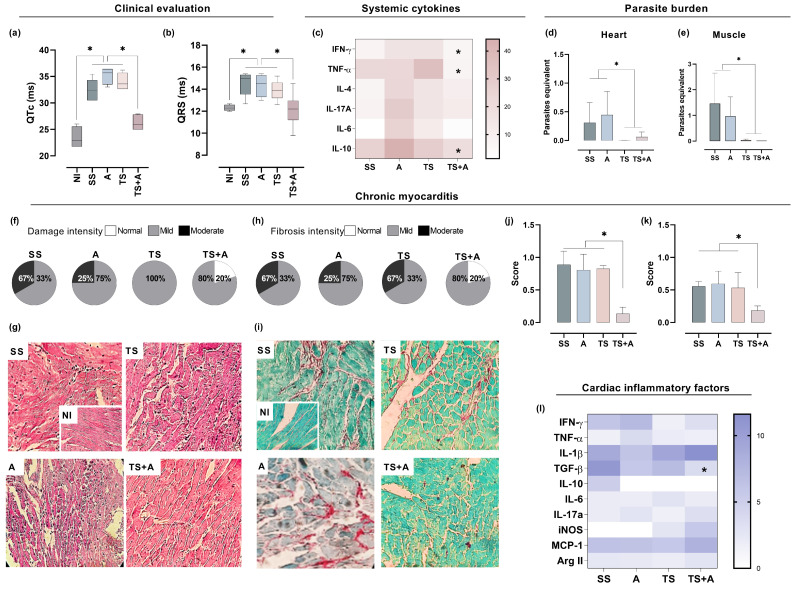
**Vaccine efficacy in the chronic phase after 111 days post-infection.** Evaluation of cardiac function by ECG: (**a**) QT interval, (**b**) QRS complex, both in milliseconds. (**c**) Heatmap of plasma cytokines. (**d**,**e**) Parasite burden by qPCR. Chronic myocarditis: infiltrates and fibrosis were classified as mild, moderate or severe. Pie charts show the relative proportion of each type of inflammatory infiltration/group (**f**) or fibrosis/group (**g**). Representative images of inflammatory infiltrations (**h**) and fibrosis (**i**) (magnification 40×). Bar graphs show the estimation of tissue damage based on the number and intensity of inflammatory infiltrations (**j**) or fibrosis (**k**). Heatmap showing heart cytokine expression in vaccinated and chronically infected animals (**l**). * *p* < 0.05.

To evaluate the impact of immunization on *T. cruzi*-induced changes in chronic cardiac function, ECG recordings were performed in anesthetized mice after 111 days post-infection. The evaluation of cardiac function revealed an increase in the magnitude of the QRS complex and the length of the QTc interval in the SS, TS and A groups compared to the TS+A and NI groups (Figure 3a,b). Notably, both parameters were preserved in the TS+A group, with values comparable to those recorded in the NI group (Figure 3a,b). Furthermore, the proportion of animals exhibiting arrhythmias differed among the groups (n = 5–6/group); 50% to 66.6% of animals in the SS, TS and A groups had rhythm abnormalities, whereas this proportion was reduced to 40% in the TS+A group. 

After euthanasia, we analyzed systemic and cardiac cytokine levels, tissue parasite load, cardiac infiltrates and fibrosis. The results revealed that TS+A immunized animals exhibited significant reductions in IL-10, TNF-α and IFN-γ levels during the chronic phase when compared to the other groups (Figure 3c). No significant differences were found in IL-4, IL-6, IL-2 and IL-17A levels among the groups (Figure 3c). Additionally, the parasite burden in the heart and skeletal muscle samples was significantly lower in the TS+A and TS groups compared to the SS and A groups (Figure 3d,e).

To assess the impact of the vaccine formulations on *T. cruzi*-induced cardiac inflammation, we examined leukocyte infiltrates in cardiac tissue using H&E-stained sections. Mice immunized with TS+A exhibited a higher proportion of normal cardiac tissue (Figure 3f,g), whereas myocarditis scores were significantly elevated in mice from the SS, TS and A groups compared to those in the TS+A group (Figure 3f). A similar trend was also observed in skeletal muscle tissue (Supplementary Figure S1).

Additionally, heart cytokine expression in TS+A immunized mice showed a significant reduction in TGF-β compared to the SS group (TS+A vs. SS, *p* < 0.05), while no significant differences were observed in other cytokines among the groups (Figure 3j). Long-term infection with *T. cruzi* can lead to substantial fibrosis in cardiac tissues, as indicated by collagen fiber accumulation (Rossi et al., 2003 [19]). To evaluate collagen deposition, we analyzed picrosirius red-stained sections of heart tissues from chronically infected mice. Figure 3i,j illustrates that TS+A mice exhibited significantly reduced collagen deposition compared to the other groups. A similar reduction in collagen deposition was observed in the skeletal muscle samples (Supplementary Figure S2). Collectively, these findings suggest that the TS+A vaccine formulation effectively reduces inflammatory cell infiltration into cardiac tissue, modulates the inflammatory response and prevents the pathology associated with chronic *T. cruzi* infection.

## 4. Discussion

In this study, we performed an in-depth immunological analysis to assess how mucosal administration of a prophylactic vaccine based on the N-terminal fragment of recombinant TS protects mice from oral *T. cruzi* infection. Our evaluation not only measured immunogenicity and vaccine efficacy during the acute phase of infection, but also examined its long-term impact on the development of Chagas cardiomyopathy, which is a key manifestation of Chagas disease. Moreover, understanding the downstream signaling pathways activated by the recombinant TS fragment combined with c-di-AMP at the nasal mucosa and their role in mediating both oral and systemic immunity is crucial for elucidating how this vaccine formulation enhances anti-parasitic responses.

An effective approach for managing oral acute infection involves increasing both mucosal but also systemic TS-specific antibody production, and simultaneously, enhancing the cellular immune response. Given that the buccal mucosa, oropharynx and palate serve as entry points for *T. cruzi,* when parasites are deposited in the oral cavity [12], the presence of specific IgA in these surfaces can help prevent these parasites from adhering to and penetrating the epithelium. The elevated levels of TS-specific IgA observed at the immunization site in the TS+A group highlight the formulation’s effectiveness in eliciting specific mucosal immunity, which may be crucial for controlling the early stages of oral infection. On the other hand, the systemic elevation of IgG2a and IgG1 indicates the development of a mixed Th1/Th2 immune profile, similar to what was observed with the C-terminal fragment of TS formulated with c-di-AMP [14]. Native active TS has been shown to skew the Th1/Th2 balance towards a Th2 response by reducing the production of IL-2, IL-13 and IFN-γ while increasing IL-4 levels, being that this mechanism is partly involved in the SAPA-driven parasite evasion of the immune response [34,35]. However, in our study, nasal administration of the TS fragment lacking the SAPA domain did not significantly elevate IL-4 levels in the NALT, though TS+A did increase IL-4 levels in plasma. Moreover, the cytokine profile triggered by TS+A is characterized by the presence of IFN-γ and IL-17A mRNA in the NALT and plasma. Similarly, intranasal immunization with a vaccine formulated with Tc52 (an antigen unrelated to the TS family) and c-di-AMP also induced IFN-γ and IL-17A secretion in T lymphocytes upon ex vivo re-stimulation [36]. These compartmentalizations of cytokine production could help to improve the anti-parasite response [37]. A cytokine environment enriched with multiple cytokines, particularly those produced by multifunctional Th1 cells involving the production of IFN-γ/IL-2/TNF-α, has been linked with vaccine protective efficacy against various protozoan parasites, such as Leishmania, as well as viruses [38] and bacteria [39]. Despite the fact that TNF-α was not detected after TS+A administration, the vaccine triggered multiple cytokines at mucosal and systemic levels, including IFN-γ and IL-2, but also Th2 (IL-4/IL-5) and Th17 (IL-17) cytokines. TS+A also prompted the production of IL-6 and IL-21, cytokines critically involved in follicular-Th (Tfh) differentiation and antibody production [40]. Numerous studies have demonstrated that c-di-AMP (as well as other dinucleotides), serve as highly effective immunopotentiators at the mucosal level, inducing a robust immune response characterized by a mixed Th1/Th2/Th17 profile [27]. This cytokine profile aligns with what we observed in our study and mirrors findings from other intranasally administered vaccines against *T. cruzi* combining recombinant peptides with c-di-AMP, such as those containing a C-terminal fragment of TS [18] or Tc52 [41]. Further supporting this, recent research with a subunit vaccine containing Ag85Bc plus c-di-AMP, administered intranasally to a mouse model of persistent *M. tuberculosis* H37Ra infection, also elicited a significantly enhanced Th1/Th2/Th17 response in the lungs and a reduction in pathological lesions [42]. Moreover, the p7 epitope, which is restricted by MHC-II and located in the N-terminal region of TS, has been associated with the induction of Th1 and Th17 lymphocytes in *T. cruzi* infection [43]. However, this epitope is absent in the fragment used in this study, as it is located between amino acids 23 to 43, while our fragment spans from 63 to 346. Taken together, these findings indicate that c-di-AMP, rather than the specific antigen, may be primarily responsible for driving this mixed cytokine pattern [27,44,45]. Additionally, the systemic and mucosal production of IL-17 may be partly mediated by TS-specific B lymphocytes, as demonstrated by Bermejo et al. in the context of *T. cruzi* infection [46]. Future studies involving various recombinant TS fragments adjuvanted with c-di-AMP will help elucidate the relative contributions of IL-17-producing Th17 and B cell populations in the vaccine’s effectiveness.

The enhanced control of the parasite achieved with the TS+A vaccine after oral infection not only improved the clinical manifestations of the disease but also effectively collaborated in the prevention of tissue damage during the acute phase of infection. This is evidenced by the nearly baseline levels of CK, GOT and GPT, along with minimal acute myocarditis, observed in vaccinated animals. TNF-α is implicated in inflammatory cell trafficking and local tissue damage [47]; therefore, reduced plasma and heart TNF-α levels during the acute phase in the TS+A infected group are notably associated with cardiac protection. Moreover, other inflammatory cytokines were also diminished in hearts of TS+A acutely-infected mice, like TGF-β, INOS and MCP-1, supporting the histological findings.

Notwithstanding the fact that our mucosal vaccine significantly diminished parasite load, attaining sterilizing immunity via vaccination could be a significant challenge. Given that the parasite can manipulate immune mechanisms for its benefit, understanding more profoundly the immune pathways triggered in intranasal vaccinated individuals may yield important insights for enhancing parasite management. Therefore, future innovative approaches are essential for boosting mucosal vaccine effectiveness.

Despite this, the vaccine’s protective effect persisted into the chronic phase, with TS+A-immunized animals showing fewer histological and electrical abnormalities, along with a reduced expression of TGF-β mRNA. In the context of pre-clinical and clinical studies of chronic Chagas disease, evidence indicates that TGF-β stimulates heart fibrosis and cardiac remodeling, and impairs heart conduction, contributing to a worse prognosis, while TGF-β inhibitors have been shown to reverse these effects [48,49], suggesting that the TS+A-induced reduction of this cytokine plays a crucial role in reducing fibrosis and supporting cardiac recovery. In addition, although chronic murine myocarditis may not perfectly mirror human chronic Chagas cardiomyopathy, BALB/c mice infected with Tulahuen exhibit clear electrocardiographic alterations, with QTc interval prolongation serving as a key indicator of histological damage [50]. This justifies the use of ECG to assess the extent to which vaccine formulations can prevent or mitigate chronic myocarditis. Our results showed that while animals in the SS, TS and A groups exhibited alterations in the QRS complex and QTc interval, those immunized with TS+A had values approaching those of the non-infected (NI) group. Additionally, the lower incidence of arrhythmias in the TS+A group suggests that this vaccine formulation effectively attenuates electrical conduction and repolarization abnormalities, particularly in the QTc interval. Prolonged QTc can indicate abnormal ventricular repolarization, which may result from cardiac autonomic dysfunction or inflammatory processes leading to fibrosis and myocardial dilation. Additionally, the administration of this formulation attenuated tissue lesions during the chronic phase of the experimental infection, which was associated with a decrease in abnormal electrical phenomena.

In this study, significant clinical effects were seen only when the selected TS fragment was combined with the c-di-AMP. However, it is important to consider several factors when evaluating the potential of this vaccine candidate. The variability among *T. cruzi* strains and the complexity of the parasite’s life cycle might still pose challenges. The infective strain used in this study, Tulahuen, predominantly affects reticulocytes but also has myotropic properties and belongs to DTU VI. This lineage is known in South America for its association with myocarditis, along with DTU V [51,52,53]. Although the choice of Tulahuen was justified by its capacity to induce cardiac damage following oral infection [54], further research is needed to evaluate vaccine protection against other strains or DTUs, particularly those more frequently linked to oral infection outbreaks, such as DTU I and IV [55,56,57]. Despite that, the high sequence identity and conservation of antigenic regions within TS-GI proteins among different DTUs support their potential as broad-spectrum vaccine candidates [15]. Additionally, studies should explore the use of metacyclic trypomastigotes and assess the effectiveness of intranasal immunization against systemic challenges that better reflect vector-borne infection, such as subcutaneous or intradermal models. Additionally, the effectiveness of adjuvants like c-di-AMP needs to be validated across diverse populations and in different experimental models to ensure broad applicability. Further research should focus on evaluating how these findings translate to real-world scenarios and whether the observed protection is effective against various *T. cruzi* strains and at different stages of the disease. Additionally, assessing the long-term efficacy and safety of the vaccine through clinical trials will be crucial for its potential application in preventing Chagas disease.

## 5. Conclusions

The data presented here demonstrates that the intranasal administration of a vaccine formulated with TS and c-di-AMP (TS+A) is highly immunogenic, triggering a mixed cytokine pattern and providing significant protection against *T. cruzi* infection. This protection is evidenced by a reduced parasite load, lessened tissue damage and attenuated acute myocarditis. Notably, the benefits of TS+A extend into the chronic phase of infection, resulting in a marked reduction in chronic myocarditis, fibrosis and functional electrocardiographic abnormalities. These findings indicate the potential to develop a mucosal vaccine against *T. cruzi* that can attenuate the progression of Chagas cardiomyopathy, the hallmark of Chagas disease.

## Data Availability

The data supporting the findings of this study are available from the corresponding author upon reasonable request.

## Supplementary Materials

The following supporting information can be downloaded at: https://www.mdpi.com/article/10.3390/vaccines12101171/s1, Figure S1: Histopathological examination of skeletal muscle at day 17 pi; Figure S2: Histopathological examination of skeletal muscle at day 111 pi. Table S1: Primer sequences used for RT-qPCR.
