# Supplementary material for: Intranasal Trans-Sialidase Vaccine Mitigates Acute and Chronic Pathology in a Preclinical Oral Chagas Disease Model
Source: Vaccines (Basel). 2024 Oct 15;12(10):1171. doi: 10.3390/vaccines12101171 (PMC11511307; doi:10.3390/vaccines12101171)
Supplement: Supplementary file 1 [file vaccines-12-01171-s001.zip › vaccines-3233644-supplementary.pdf]

vaccines

Gene	Forward (5′-3′)	Reverse (5′-3′)
IFN-γ	AGACAATCAGGCCATCAGCAAC	CTCATTGAATGCTTGGCGCTG
IL-17a	CAAAGCTCAGCGTGTCCAAA	CTTCCCAGATCACAGAGGGATA
IL-4	GCCAAACGTCCTCACAGCAA	TCTGCAGCTCCATGAGAACACT
IL-2	CCTGAGCAGGATGGAGAATTACA	CCTGAGCAGGATGGAGAATTACA
IL-6	GAGGATACCACTCCCAACAGACC	AAGTGCATCATCGTTGTTCATACA
L-5	AGCACAGTGGTGAAAGAGACC	TTATGAGTAGGGACAGGAAGCC
IL-21	AGAAGGCCAAACTCAAGCCA	CATACGAATCACAGGAAGGGCA
TNF-a	CACAGAAAGCATGATCCGCGAC	GAACTTCTCATCCCTTTGGGGAC
IL-1b	AGACAACTGCACTACAGGCTC	TTGTCGTTGCTTGGTTCTCCT
TGF-b	TGACGTCACTGGAGTTGTACGG	GGTTCATGTCATGGATGGTGC
IL-10	CAAGCCTTATCGGAAATGATCCA	CCTTGTAGACACCTTGGTCTTG
iNOS	GACGAGACGGATAGGCAGAGATTG	CCTGGGAGGAGCTGATGGAGTAG
MCP-1	GGCTCAGCCAGATGCAGTTAA	CCTACTCATTGGGATCATCTTGCT
Arg II	TGATTGGCAAAAGGCAGAGG	CTAGGAGTAGGAAGGTGGTC
GAPDH	AGCAATGCATCCTGCACCACCA	ATGCCAGTGAGCTTCCCGTTCA

Table S1: Primer sequences used for RT-qPCR.

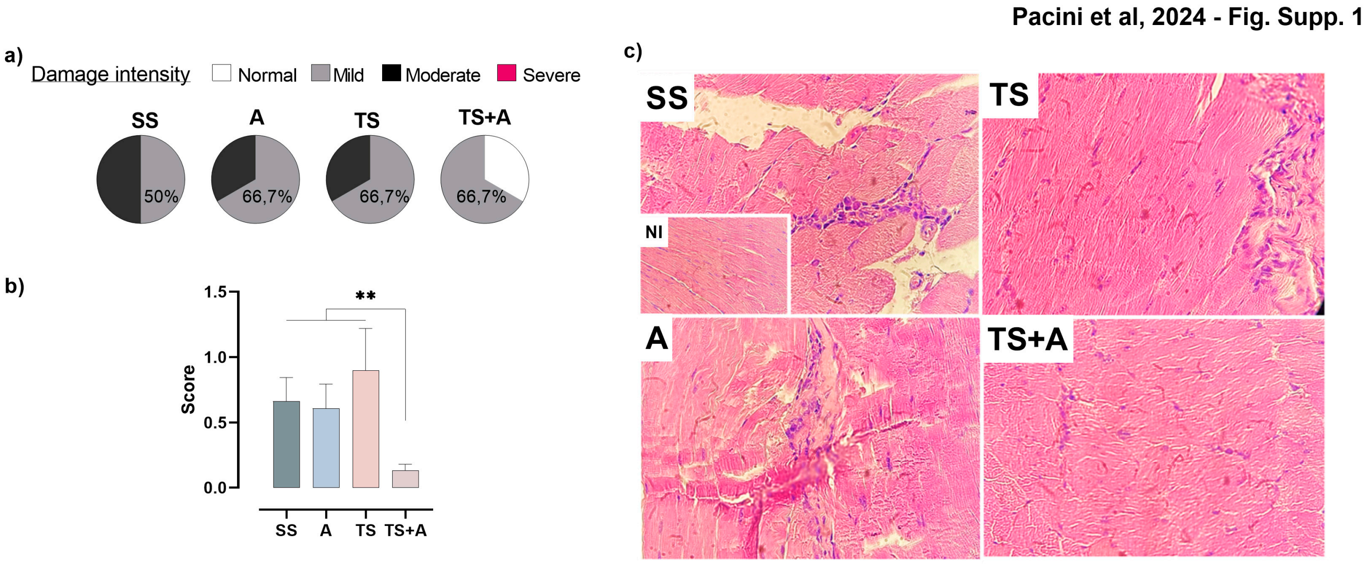

Figure S1: Histopathological examination of skeletal muscle at day 17 pi. Histological sections of 5 µm were stained with hematoxylin/eosin **a**) Infiltrate intensities were classified as mild, moderate, or severe. Pie charts illustrate the proportion of animals within each group that exhibited each type of inflammatory infiltrate. **b**) The inflammatory score estimates the degree of tissue damage in each group based on the number and intensity of inflammatory infiltrates. **c)** Representative images of tissues from immunized and orally infected animals. The smaller image inserted in the photograph corresponding to the SS group shows a normal tissue from an uninfected and unimmunized animal (40X magnification). **p<0.001.

.

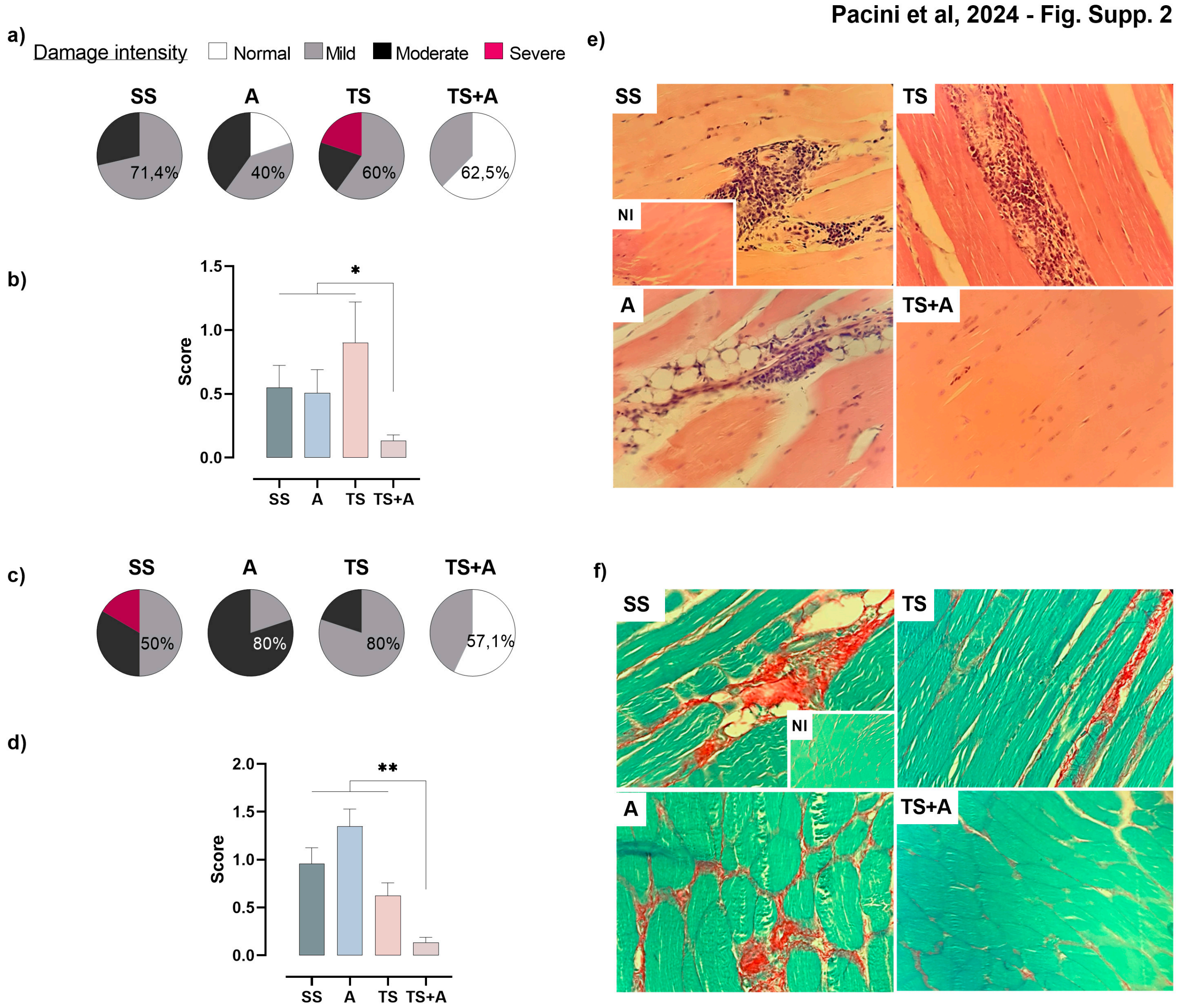

Figure S2: Histopathological examination of skeletal muscle day at 111 pi. Histological sections of 5 μm were stained with hematoxylin/eosin and picrosirius red. Chronic infiltrates and fibrosis were classified as mild, moderate, or severe. Pie charts show the relative proportion of each type of inflammatory infiltrate/group (**a**) or fibrosis (**c**). Bar graphs show the estimation of tissue damage based on the number and intensity of inflammatory infiltrates (**b**) or fibrosis (**d**). Images show representative histological findings in the muscle tissue of immunized and orally infected animals: inflammatory infiltrates (**e**) and fibrosis (**f**). The smaller images inserted in the SS group show normal muscle tissues from uninfected and non-immunized mice (magnification 40X) *$p<0.05$ **$p<0.001$.
